# The effect of medial longitudinal arch height and medial longitudinal arch support insoles on postural balance in perimenopausal women

**DOI:** 10.3906/sag-1808-39

**Published:** 2019-06-18

**Authors:** Levent KARATAŞ, Doğa VURALLI, Zafer GÜNENDİ

**Affiliations:** 1 Gazi University Faculty of Medicine, Department of Physical Medicine and Rehabilitation, Ankara Turkey; 2 Gazi University Faculty of Medicine, Department of Neurology and Algology, Ankara Turkey

**Keywords:** Arch support insole, pes planus, low medial longitudinal arch height, postural balance, perimenopausal women

## Abstract

**Background/aim:**

Changes in balance and postural control have been reported during the perimenopausal period. We investigated the effect of medial longitudinal arch height and medial arch support insoles on postural sway and balance in middle-aged perimenopausal women.

**Materials and methods:**

29 women with normal arches and 29 women with low arches were included in the study. The foot arches of the participants were determined using the arch height index. The static balance index (SBI) measured by Kinesthetic Ability Trainer 3000 and functional reach test were used to evaluate postural balance. Measurements were obtained from all participants with and without medial arch support insoles.

**Results:**

The SBI-total scores without the insoles were found to be significantly higher in the lower arch group than in the normal arch group. SBI-total, SBI-anteroposterior, and SBI-mediolateral scores significantly improved in the low arch group in the presence of insoles, whereas the usage of insoles resulted in no difference in the normal arch group. In the presence of insoles, the reach distances to left and right sides increased in both groups, while the forward functional reach distances decreased.

**Conclusion:**

Medial longitudinal arch height and medial arch support insoles affect the balance parameters in perimenopausal women.

## 1. Introduction

Pes planus is defined as a decrease in medial longitudinal arch (MLA) height, hyperabduction of the forefoot, and valgus posture of the midfoot and hindfoot. Pes planus is known to be associated with spine and lower extremity pain (1). Additionally, arch abnormalities can also affect balance. Plantar skin, ligaments forming the foot arches, sensory inputs from mechanoreceptors located in intrinsic and extrinsic foot muscle tendons and joint capsules, and flexibility and stability of foot arches are closely related to standing and walking balance (2). Although the exact cause is unknown, a low MLA negatively affects balance by disrupting the stability of the foot and the relation between the foot and the floor (3–6). There are studies in the literature which report a relationship between arch pathologies and poor postural sway in young adults and seniors over 65 years of age (3,6,7). The ability to maintain balance diminishes with advancing age. It has been reported that the frequency of falls in middle and advanced ages is higher in women (8). 

Hormonal changes, especially in the perimenopausal period, negatively affect postural control by their influence on central nervous system (9–10). However, there has been no study on perimenopausal women that investigates the relationship between balance and foot arch abnormalities. We hypothesized that decrease in medial longitudinal arch height (pes planus) impairs balance and postural control, and medial arch support insoles improve balance and postural control in perimenopausal women. Therefore, in our study, we investigated the effect of medial longitudinal arch height and medial arch support insoles on the postural balance of perimenopausal women. 

## 2. Materials and methods

### 2.1. Study design

Subjects were consecutively recruited from the Physical Medicine and Rehabilitation outpatient clinic between January 2016 and June 2016. Inclusion criteria were 1) female subjects, 2) subjects in the perimenopausal period (having irregular periods or less than one year passed after the last menstruation), 3) subjects who could remain on both left and right feet for at least 15 s, 4) no pain associated with lower extremities or spine. Exclusion criteria were 1) decreased range of motion in spine and peripheral joints, 2) history of surgery or trauma, 3) balance disorders, 4) presence of vertigo, 5) presence of any central or peripheral nervous system disease, 6) female subjects with a history of early or late menopause. Two subjects were excluded from the study. A 38-year-old subject had a history of early menopause and a 57-year-old subject had a history of late menopause; therefore, they were excluded from the study.

**Figure 1:**
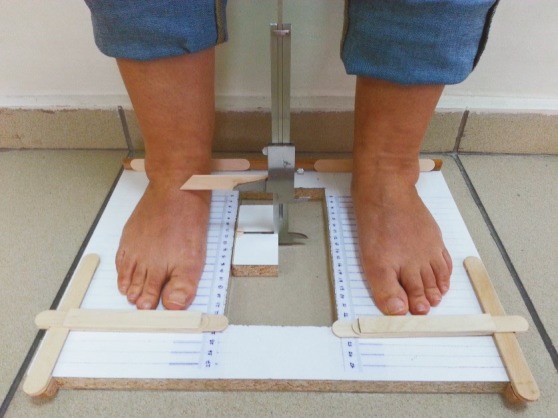
The evaluation of the medial longitudinal arch with the arch height index method.

Footprint methods are commonly used to evaluate medial longitudinal arch morphology. Footprint methods may be influenced by foot sole soft tissue; therefore, new methods have been investigated to achieve more reliable measurements. The arch height index is a more reliable method for evaluating medial longitudinal arch morphology. First, the foot length, which is the distance between the most posteriorly projecting point on the heel to the tip of the most anteriorly projecting toe, is measured. The distance from the most posteriorly projecting point on the heel to the dorsal prominence of the first metatarsophalangeal joint is then measured and is noted as the truncated foot length. At 50% of the foot’s length, the vertical distance from the floor to the foot dorsum is accepted as arch height. The arch height index is the ratio of arch height to the truncated foot length. Perimenopausal women aged between 40–55 years who agreed to participate in the study were evaluated with arch height index (AHI) (Figure 1). The participants who had AHI lower than 0.34 during bipedal stance were included in the low MLA group (11–14). Subjects who had normal medial arch according to AHI measurement were included in the control group. We calculated 29 subjects in each group to achieve a difference of 55 ± 101 in SBI between low and normal arch groups (power = 0.80, α = 0.05) (15). All of the participants gave their written informed consent before the study. The local ethics committee approved the study (E-15-171). Demographic features, systemic diseases, leg dominance, and musculoskeletal examination of the subjects were recorded. The ball kicking test was used to determine leg dominance (16). In the ball kicking test, the subject was asked to kick a ball with moderate intensity and the preferred leg to kick the ball was determined as the dominant leg.

### 2.2. Balance measurements

**Figure 2:**
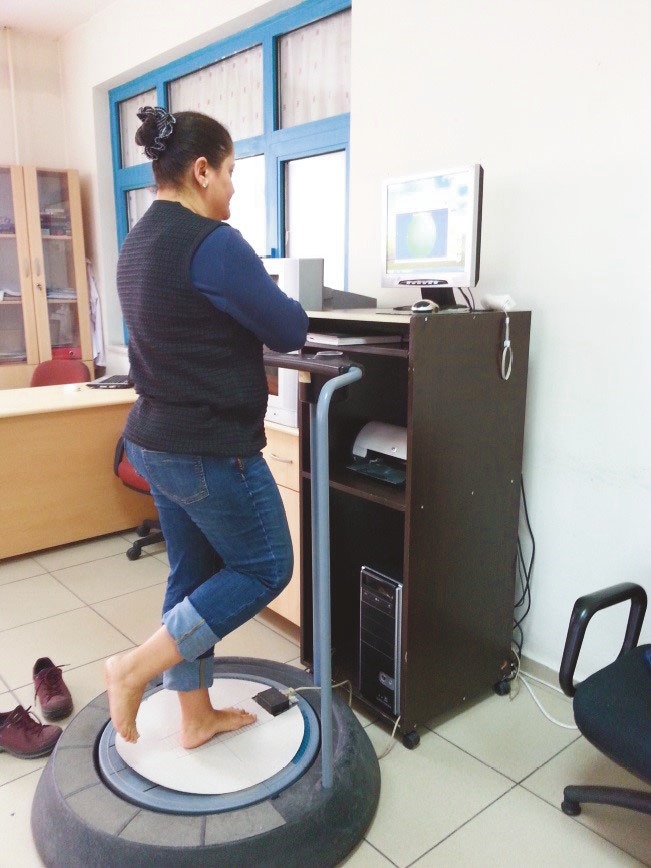
The measurement of the static equilibrium index with the Kinesthetic Ability Trainer 3000.

Static balance index (SBI) and functional reach test (FRT) were used to evaluate postural balance (17,18). SBI was measured with a Kinesthetic Ability Trainer 3000 (KAT 3000, Berg, Vista, CA, USA). Subjects stood on one bare foot, with arms crossed on the chest, and looked at the X sign on the screen during the measurement (Figure 2). SBI was evaluated for both sides. The pound per square inch (PSI) level was adjusted to 7 to maintain platform stability. After a trial period of 15 s, 3 measurements, each lasting 15 s, were taken with 3 min resting period in between. Mediolateral (ML), anteroposterior (AP), and total SBI scores were recorded. The lowest ML, AP, and total SBI scores of the 3 measurements were used for the analysis. Lower SBI values suggested better postural balance, whereas higher SBI values reflected impaired postural balance and increased sway.

Functional reach test was performed during a bipedal stance on a hard floor with bare feet, 10 cm between the heels and 12 cm between the first toes. Subjects were first asked to reach as far as they could, forward and then to left and right sides without taking a step. After completing 3 successful trials, the furthest distances in each direction were used for analysis. 

To investigate the effect of insoles on balance, SBI measurement and FRT were performed for all participants with and without the insoles. Measurements with and without insoles were done on the same day, with a 15 min rest period in between the measurements. Ready-to-wear medial arch support cork insoles were used in the study. To eliminate the learning effect associated with repeated measurements, half of the subjects were randomly selected to be evaluated first with the insoles and the other half without the insoles in both groups. 

### 2.3. Statistical analysis

Categorical variables were compared using a chi-square test. Normality assessment was performed with a Shapiro–Wilk test. Comparisons between independent samples were done using Student’s t-test for normally distributed numerical variables and Mann–Whitney U test for nonnormally distributed variables. Comparisons between dependent groups were evaluated using a paired t-test for parametric data and the Wilcoxon signed rank test for nonparametric data. SPSS for Windows version 23.0 was used for all statistical analysis. 

## 3. Results

### 3.1. Demographic and baseline characteristics

Demographic features of the participants are shown in Table 1. There was no significant difference between low and normal arch groups regarding age, height, weight, body mass index, leg dominancy, other foot deformities, and comorbid diseases. The majority of the subjects had right leg dominancy (93% in the low arch group and 100% in the normal arch group). 

**Table 1:** Demographic features and comorbidities of the participants.

	Low arch group(n = 29)	Normal arch group(n = 29)	P
Age (year) (mean ± SD)	48.8 ± 4.7	49.6 ± 4.3	0.504
Height (cm) (mean ± SD)	158.8 ± 5.8	159.4 ± 7.3	0.720
Weight (Kg) (mean ± SD)	73.0 ± 10.4	68.6 ± 9.7	0.098
BMI (mean ± SD)	29.0 ± 4.1	27.1 ± 4.5	0.106
Dominant leg (right/left)	27/2	29/0	0.492
Diabetes mellitus	3/29 (10%)	2/29 (7%)	0.640
Thyroid disorders	8/29 (28%)	7/29 (24%)	0.764
Hallux valgus	1/29 (3%)	1/29 (3%)	1.000
Other systemic diseases	15/29 (52%)	17/29 (59%)	0.597

BMI: body mass index; cm: centimeter; kg: kilogram; SD: standard deviation.

Mean AHI for the low arch group was 0.313 ± 0.016 for the right foot, 0.310 ± 0.018 for the left foot. Mean AHI for the normal arch group was 0.364 ± 0.020 for the right foot, 0.362 ± 0.017 for the left foot. No difference was observed between right and left AHIs in any of the participants.

### 3.2. Postural balance parameters

Functional reach distance (FRD) and SBI scores obtained during the measurements without insoles are shown in Table 2. In the low arch group, mean SBI total score measured without insoles for both feet was significantly higher when compared with the normal arch group. Forward FRD and FRD to right and left sides were statistically comparable when they were measured without insoles. The effect of insoles on the test results are shown in Tables 3 and 4. In the measurements with insoles, a prominent decrease in the total, mediolateral, and anteroposterior SBI scores were observed in the low arch group, whereas no difference was found in the normal arch group. In the presence of insoles, right and left FRDs markedly increased while forward FRD decreased in both groups. There was no difference between groups regarding absolute and percentage changes in FRDs. 

**Table 2:** Comparison of static balance index and functional reach distances between groups.

	Low arch group (n = 29)		Normal arch group (n = 29)		p
	Mean ± SD	Median (min–max)	Mean ± SD	Median (min–max)	
Right SBI-total	168.2 ± 59.1	166 (58–296)	127.1 ± 50.3	123 (34–253)	0.006
Right SBI-AP	154.4 ± 59.3	15058–296)	127.1 ± 58.9	123 (34–275)	0.080
Right SBI-ML	167.4 ± 58.5	166 (58–296)	126.7 ± 49.6	123 (34–253)	0.005
Left SBI-total	180.6 ± 72.8	169 (79–386)	133.6 ± 60.5	121 (55–309)	0.007
Left SBI-AP	171.4 ± 75	165 (79–386)	132.9 ± 78.5	113 (42–435)	0.017
Left SBI-ML	180.9 ± 72	169 (92–386)	139.3 ± 83.3	121 (55–483)	0.005
FRD-forward (cm)	28.2 ± 3.9	29 (18–34)	28.3 ± 4.8	28 (17–38)	0.952
FRD-right (cm)	15.8 ± 2.8	16 (10–24)	16.0 ± 3.2	16 (10–22)	0.776
FRD-left (cm)	16.3 ± 2.5	16 (10.5–21)	16.0 ± 3.1	16 (10–21)	0.745

SBI: static balance index; FRD: functional reach distance; AP: anteroposterior; ML: mediolateral; cm: centimeter; SD: standard deviation.

**Table 3:** The effect of insoles on balance parameters in women with low arches.

Low arch Group	Without insoles		With insoles		P
	Mean ± SD	Median (min–max)	Mean ± SD	Median (min–max)	
Right SBI-total	168.2 ± 58.4	162 (58–296)	120.5 ± 42.9	119 (34–212)	<0.001
Right SBI-AP	154.4 ± 59.3	148 (58–296)	119.8 ± 45.1	119 (4–212)	<0.001
Right SBI-ML	167.4 ± 58.5	162 (58–296)	122.8 ± 44	118 (34–211)	<0.001
Left SBI-total	180.6 ± 72.8	169 (79–386)	128.2 ± 64.7	107 (46–277)	<0.001
Left SBI-AP	171.4 ± 75.0	162 (79–386)	122.2 ± 46.3	104 (35–277)	<0.001
Left SBI-ML	180.9 ± 72.0	166 (92–386)	122.8 ± 44.1	107 (46–276)	<0.001
FRD-forward (cm)	28.2 ± 3.9	29 (18–34)	26.9 ± 3.6	28 (20–33)	0.018
FRD-right (cm)	15.8 ± 2.8	16 (10–24)	16.6 ± 2.5	16.25 (11–22)	0.002
FRD-left (cm)	16.3 ± 2.1	16 (10–21)	17.4 ± 2.8	17 (12–22)	<0.001

SBI: static balance index; FRD: functional reach distance; AP: anteroposterior; ML: mediolateral; cm: centimeter, SD: standard deviation.

**Table 4:** The effect of insoles on balance parameters in women with normal arches.

Normal arch group	Without insoles		With insoles		P
	Mean ± SD	Median (min–max)	Mean ± SD	Median (min–max)	
Right SBI-total	127.1 ± 50.3	123 (34–253)	123.9 ± 56.8	125 (16–246)	0.675
Right SBI-AP	127.1 ± 58.9	123 (34–275)	124.1 ± 57.0	125 (16–247)	0.696
Right SBI-ML	126.7 ± 49.6	123 (34–253)	121 ± 57.1	125 (16–246)	0.506
Left SBI-total	133.6 ± 60.5	121 (55–309)	132.7 ± 59.4	121 (40–227)	0.814
Left SBI-AP	132.9 ± 78.5	113 (42–435)	131 ± 58.9	113 (40–227)	0.959
Left SBI-ML	139.3 ± 83.3	121 (55–483)	127.7 ± 61.9	120 (23–227)	0.666
FRD-forward (cm)	28.3 ± 4.8	28 (17–38)	26.1 ± 5.1	26 (16–35)	0.001
FRD-right (cm)	16.0 ± 3.2	16 (10–22)	16.9 ± 2.9	17 (12–22)	0.013
FRD-left (cm)	16.0 ± 3.1	16 (10–21)	17.03 ± 2.7	17 (13–23)	0.001

SBI: static balance index; FRD: functional reach distance; AP: anteroposterior; ML: mediolateral; cm: centimeter; SD: standard deviation.

## 4. Discussion

In this study, we showed that low MLA has a negative effect on SBI in perimenopausal women. The use of arch support insoles in the patients with low MLA improved SBI; however, it did not cause any improvement in SBI in the normal MLA group. Additionally, arch support insoles increased mediolateral FRD in both arch groups. 

In the low MLA group, SBI-total, SBI-ML, and SBI-AP scores without the use of insoles were higher when compared to the normal MLA group. Several studies in the literature have shown a relationship between low MLA and poor postural stability. However, the results of the previous studies cannot be generalized to perimenopausal women because they were conducted using different age groups. Saghazadeh et al. and Anzai et al. have shown a relation between low MLA and poor postural stability in patients over 65 years of age (6,7). In this age group, it was shown that as MLA height decreased, mediolateral postural sway increased (7). On the contrary, in young adults, pes planus had no impact on postural balance (4,19). In this study, we examined the effects of MLA height and MLA support insoles on postural balance in perimenopausal women since estrogen withdrawal is associated with increased fall frequency and mediolateral postural stability is impaired during the perimenopausal period (10). As a result, we found that low MLA had a negative effect on postural balance in perimenopausal women.

In our study, the use of insoles improved total, mediolateral, and anteroposterior SBI scores in patients with low arches. There are several possible explanations for the positive impact of MLA support insoles on balance. Insoles decrease foot pronation by increasing the stability of the joints of the foot, modulate sensory stimuli from the sensory receptors of the plantar skin, improve joint position sense, and alter the tonus of the muscles which support the foot arch. Amelioration of postural balance on the single leg stance in patients with ankle inversion injury has been reported with the use of insoles (20,21). The relation between balance and insoles which affect plantar sensation is not clear. Vibrating insoles and facilitatory insoles with a raised ridge around the perimeter have been shown to enhance balance in older adults with decreased plantar sensation. However, in healthy young adults, similar results have not been observed (22,23). The limitation of the study was that they did not consider the MLA morphology of the participants.

Some studies have suggested that MLA support insoles had no effect on balance during a single leg stance. However, these studies were done in young adults. Tahmasebi et al. showed no significant change in the center of pressure distance with the use of MLA support insoles in 15 young adults with pes planus and 15 healthy controls. Similarly, Payehdar et al. showed that rigid or semirigid insoles which have support below the subtalar joint had no effect on postural sway scores in 20 young patients with moderate or severe pes planus (24,25). We included perimenopausal women in this study since hormonal changes are thought to affect postural balance during the perimenopausal period. As a result, we showed acute improvement in postural balance with MLA support insoles in perimenopausal women with pes planus. 

In this study, MLA support insoles increased FRDs to right and left side while it decreased forward FRD in all subjects. This is the first study to investigate the effect of the use of arch support insoles on FRD. FRT aims to measure the stability limits of the body’s center of gravity. Medial arch support insoles increased the stability limit in all subjects regardless of the medial arch height. In contrast to this positive impact in the mediolateral direction, insoles decreased forward FRD, possibly due to alteration in plantar fascia tension. Lewinson et al. reported that custom-made insoles decreased vertical loading rate on plantar fascia in healthy subjects (26). Alteration in plantar fascia tension due to decreased vertical loading could probably affect forward FRD. Future studies would be required to clarify this issue. 

The strengths of our study are as follows: 1) this is the first study to investigate the effect of MLA height and MLA support insoles on postural balance in perimenopausal women, 2) for the first time in the literature we showed the positive effect of insoles on FRD in the mediolateral direction, 3) the use of the arch height index instead of the footprint method in the examination of arch height prevented the confounding effect of foot sole soft tissue, 4) additionally, to prevent the confounding effect of different shoes on the results, measurements were done with bare feet, 5) to eliminate the learning effect during repeated measurements with KAT (27), half of the subjects in both groups were randomly selected to be first evaluated with insoles and the other half without insoles.

The limitations of the study are as follows: 1) we only evaluated static balance and for this reason we cannot state an opinion about the effect of pes planus and the use of MLA support insoles on dynamic balance in perimenopausal women, 2) we did not investigate the long term effects of insoles on postural balance, 3) we did not evaluate the hormonal status of the subjects. 

In conclusion, low MLA height had a negative effect on postural balance in perimenopausal women while MLA support insoles improved balance parameters in our study. We suggest the use of arch support insoles to improve postural control in perimenopausal women even in the absence of pain.
